# Genome Sequence of Lactiplantibacillus plantarum ATCC 202195, a Probiotic Strain That Reduces Sepsis and Other Infections during Early Infancy

**DOI:** 10.1128/MRA.00741-20

**Published:** 2020-09-24

**Authors:** Matthew E. Wright, Annabelle O. Yu, Maria L. Marco, Pinaki Panigrahi

**Affiliations:** a Department of Food Science and Technology, University of California, Davis, California, USA; b Department of Pediatrics, Division of Neonatal-Perinatal Medicine, Georgetown University Medical Center, Washington, DC, USA; Broad Institute

## Abstract

The draft genome of probiotic Lactiplantibacillus plantarum ATCC 202195, with the trademark PPLP-217, is comprised of 3,368,305 bp with a G+C content of 44.3% and no plasmids. The strain is able to grow on lactose, raffinose, and fructooligosaccharides.

## ANNOUNCEMENT

Lactiplantibacillus plantarum (previously Lactobacillus plantarum [1]) is required for the production of many fermented foods and beverages, and strains of this species are applied as probiotics intended to promote human health (2, 3). We report here the draft genome sequence of *L. plantarum* ATCC 202195, a strain isolated from the stool of an 11-month-old infant and shown to reduce sepsis, respiratory tract infections, omphalitis, and diarrhea in infants when provided early in life together with fructooligosaccharides (FOS) (4). Because no personal identifiers were collected and the lactobacilli were isolated from the discarded diapers of healthy infants, this study was waived from institutional review board (IRB) review and consenting of parents.

Lyophilized *L. plantarum* ATCC 202195 was reconstituted in de Man, Rogosa, and Sharpe (MRS) broth (BD, Franklin Lakes, NJ) and then streaked onto MRS agar for single-colony isolation. To prepare the genomic DNA, one colony of *L. plantarum* ATCC 202195 was inoculated into MRS broth and incubated at 37°C overnight. Cells were harvested by centrifugation at 5,000 × *g* for 10 min, and genomic DNA was isolated by phenol-chloroform extraction followed by RNase A (Qiagen, Germantown, MD) treatment with an ethanol precipitation (5) and quantified with a Qubit fluorometer. Next, a paired-end, whole-genome library was prepared with the Nextera DNA Flex library kit (Illumina, San Diego, CA) following the manufacturer’s instructions. The library was sequenced on an Illumina MiSeq v2 instrument, generating on average 252,195 pairs of 250-bp reads at the UC Davis Genome Center (http://dnatech.genomecenter.ucdavis.edu).

Reads were processed with Cutadapt v1.18 using default settings, followed by assembly with SPAdes v3.12.0 (using k-mers 51, 71, 91, and 121) (6, 7). QUAST v4.6.3 was used for quality assessment (8). The assembly consisted of 3.327 Mbp distributed over 20 contigs (500 bp or more) with a mean G+C content of 44.32% and a mean coverage of 32.4×. The *N*_50_ and *L*_50_ values of the assembly were 380,627 bp and 4, respectively. Annotation of the assembly was done with the NCBI Prokaryotic Genome Annotation Pipeline (v4.9), which predicted 3,138 open reading frames (ORFs) in total, including 63 tRNAs and 1,206 hypothetical genes (9). Plasmids were searched for using PlasmidFinder v2.0.1 (10); however, none were found. Two putative bacteriocins (plantaricins A and EF) were identified using the *ba*cteriocin *ge*nome mining too*l* (BAGEL) v4 (11).

*L. plantarum* ATCC 202195 was inoculated into MRS broth (12) modified to remove the beef extract and to contain 1% (wt/vol) glucose, lactose, or raffinose at a starting optical density at 600 nm (OD_600_) of 0.05. Cells reached a final OD_600_ between 2.6 and 2.8 when incubated in a Synergy 2 microplate reader (Biotek Instruments, Winooski, VT) at 37°C for 24 h. Strain ATCC 202195 also grew in MRS broth containing 1% (wt/vol) fructooligosaccharides (FOS) (Leatherhead Food International, Epsom, United Kingdom) under the same conditions to an OD_600_ of 1.0 ± 0.01. This strain also contains the genes encoding a sucrose phosphoenolpyruvate transport system (PTS) and a β-fructofuranosidase associated with FOS metabolism in *L. plantarum* WCFS1 (13).

### Data availability.

This whole-genome shotgun project has been deposited at DDBJ/ENA/GenBank under the accession number PRJNA521530. The unassembled reads are also available from the NCBI Sequence Read Archive (SRA) under the accession number SRS4344418.
